# Distribution pattern of entry holes of the tree-killing bark beetle *Polygraphus proximus*

**DOI:** 10.1371/journal.pone.0246812

**Published:** 2021-02-09

**Authors:** Shin-ya Takei, Kenta Köbayashi, Etsuro Takagi

**Affiliations:** Department of Tourism Science, Graduate School of Urban Environmental Sciences, Tokyo Metropolitan University, Hachioji, Tokyo, Japan; US Department of Agriculture, UNITED STATES

## Abstract

Bark beetles attack their hosts at uniform intervals to avoid intraspecific competition in the phloem. Bark texture and phloem thickness also affect bark beetle attacks, and the bark characteristics are not spatially homogeneous; therefore, the distribution patterns of entry holes can demonstrate an aggregated distribution. *Polygraphus proximus* Blandford (Coleoptera: Scolytinae) is a non-aggressive phloephagous bark beetle that feeds on Far Eastern firs. They have caused mass mortality in Russia and Japan. However, the distribution pattern of entry holes of *P*. *proximus* and spatial relationships with bark characteristics have not been studied. Thus, we investigated the distribution pattern of entry holes of *P*. *proximus*. The distribution of entry holes was significantly uniform in most cases. As the attack density increased, an aggregated distribution pattern within a short distance (< 4.0 cm) was observed. The rough bark had a significantly higher number of entry holes than the remaining bark. The distribution pattern of entry holes demonstrated a significantly aggregated spatial association with rough bark. Finally, rough bark around knots had significantly thicker phloem than the remaining barks. These suggest that *P*. *proximus* may preferentially attack rough bark to reproduce in the thicker phloem under a rough bark surface.

## Introduction

Most bark beetles reproduce in the phloem tissue of woody plants. Their adults land on a tree, bore into the phloem, copulate, and excavate galleries along which they oviposit. The larvae feed and develop as they construct galleries in the phloem. In the bark, a higher density results in severe intraspecific competition for nutritious phloem among larvae and low reproductive success [1–3]. Accordingly, to avoid intraspecific competition in the phloem, adults attack at uniformly, regular intervals compared to a random pattern [4,5].

Both outer and inner bark characteristics can also affect bark beetle attacks. For instance, several bark beetles preferentially attack a certain surface (i.e., outer bark) texture, such as rough texture and crevices [6–9]. Phloem (i.e., inner bark) thickness is also positively correlated with attack density [10,11] because thicker phloem has more nutrients available for egg production and brood growth than thin phloem. These positive correlations between bark characteristics and attack density may result in aggregated distribution patterns of entry holes. However, little is known regarding the mechanisms of preferential attacks on rough barks and thicker phloem.

The non-aggressive phloephagous bark beetle *Polygraphus proximus* Blandford (Coleoptera: Scolytinae) feeds on Far Eastern firs. They infest fresh-cut logs and trees weakened by fire, pathogens, typhoons, or defoliation during the endemic phase in their native range [12–14]. It has become a striking example of biological invasion in Russia. It has invaded European Russia and Western Siberia and caused rapid degradation of planted and natural forests [14–16]. In Siberia, *P*. *proximus* is the most destructive pest in natural *Abies sibirica* Ledeb. forests [14,16,17]. They also caused the mortality of *A*. *firma* Sieb. et Zucc trees in Japan [18]. Recently, mass mortalities of *A*. *veitchii* Lindl and *A*. *mariesii* Masters trees have been observed in Japan [19–21].

To date, the distribution pattern of entry holes of *P*. *proximus* has not been demonstrated. We hypothesized that rough bark had thicker phloem within a species, and the distribution pattern of entry holes of *P*. *proximus* was accordingly aggregated on the rough bark. The distribution pattern of entry holes can influence population dynamics via reproductive success in the bark [22,23]. Thus, we investigated the distribution pattern of *P*. *proximus* and the spatial association between entry holes and bark surface roughness. The aim of the present study was to determine 1) the distribution pattern of entry holes of *P*. *proximus*, 2) the spatial association between entry holes and bark roughness, and 3) the association between bark surface roughness and phloem thickness.

## Materials and methods

### Ethics statement

The following institutes granted permission of the field surveys and samplings: The University of Tokyo Hokkaido Forest, The University of Tokyo; The University of Tokyo Chiba Forest, The University of Tokyo; Sugadaira Research Station, University of Tsukuba; Yatsugatake Forest Station, University of Tsukuba; Education and Research Center of Alpine Field Science, Shinshu University.

### Bark beetle

*Polygraphus proximus* feeds on the following Far Eastern fir species: *Abies firma*, *A*. *holophylla* Maxim., *A*. *homolepis* Sieb. & Zucc., *A*. *mariesii*, *A*. *nephrolepis* (Trautv. ex Maxim.) Maxim., *A*. *sachalinensis* (Fr. Schmidt) Masters, *A*. *sibirica*, and *A*. *veitchii* [12,13,24]. They are native to northeastern China, Korea, Japan, and the southern part of the Russian Far East [12,16]. The male beetle makes an entry hole and tunnels into the bark of the host. Females then enter the entry hole to mate. While a male sex pheromone is suspected, it has never been confirmed [25,26]. Their mating system is monogyny [24,27]. The females produce double-armed horizontal mother-galleries beneath the bark for laying eggs [12,16,25]. Each offspring makes its exit hole [25,26].

### Fir species

*Abies* species are coniferous evergreen trees that grow to a height of 25–30 m. Five *Abies* species are native to Japan. *A*. *sachalinensis* is native to the Sakhalin and Kuril Islands, Russia, and Hokkaido Island, Japan. *A*. *veitchii* is native to Honshu and Shikoku, Japan. It dominates mountain forests at elevations of 1500–2500 m [28]. *A*. *mariesii* is native to the mountains of central and northern Honshu, Japan. *A*. *firma* is native to central and southern Japan. *A*. *homolepis* is native to the mountains of central and southern Honshu and Shikoku, Japan.

### Log preparation

Four to eight un-infested trees (diameter of breast height: 14–20 cm) of each of the five *Abies* species were felled in April and May 2019 (Table 1). The trees were cut into logs, each 1 m in length. To prevent them from drying, both cut-ends were coated with paraffin. Five logs from each of the five species were randomly selected and we placed them at Sugadaira Research Station, Mountain Science Center, University of Tsukuba, in Ueda City, Nagano Prefecture, Japan on May 17, 2019.

**Table 1:** Study sites and dates of *Abies* species used in this study.

Study site	Species	Collection date	Latitude / longitude
The University of Tokyo Hokkaido Forest, The University of Tokyo, in Furano City, Hokkaido, Japan (UTHF)	*A*. *sachalinensis*	April 15, 2019	43°10’N / 142° 23’E
Sugadaira Research Station, Mountain Science Center, University of Tsukuba, in Ueda City, Nagano Prefecture, Japan (SRS)	*A*. *homolepis*	April 17, 2019	36°31’N / 138°21’E
Yatsugatake Forest Station, Mountain Science Center, University of Tsukuba, in Minamisaku County, Nagano Prefecture, Japan (YFS)	*A*. *veitchii*	April 18, 2019	35°56’N / 138°28’E
The University of Tokyo Chiba Forest, The University of Tokyo, Kamogawa city, Chiba Prefecture, Japan (UTCBF)	*A*. *firma*	April 24, 2019	35°07’N / 140°09’E
Education and Research Center of Alpine Field Science, the Shinshu University, Ina County, Nagano Prefecture, Japan (AFC)	*A*. *mariesii*	May 13, 2019	35°49’N / 137°51’E

### Distribution of entry holes and rough bark around knots

**Fig 1:**
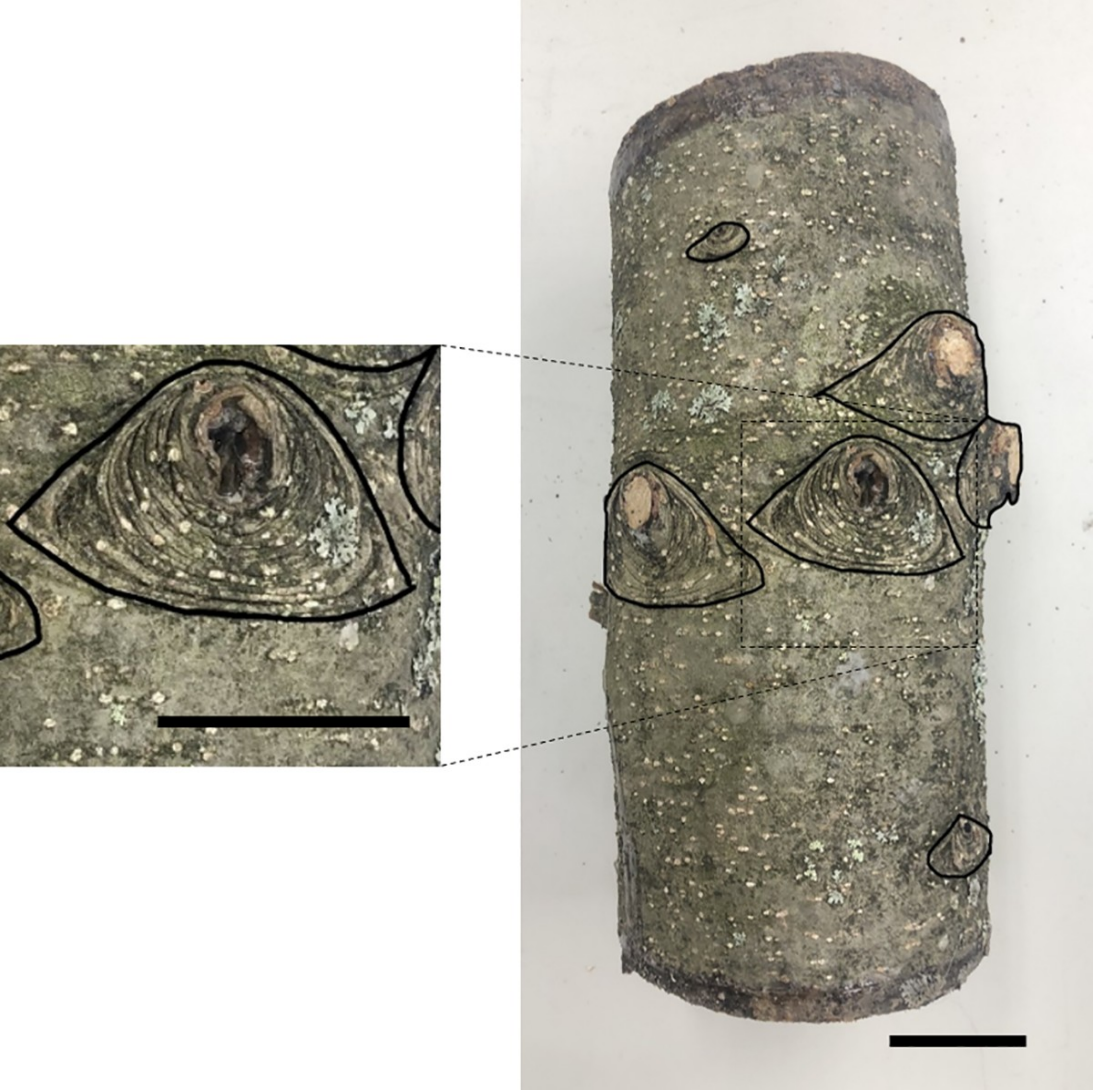
Bark of *A*. *veitchii*. The areas surrounded by black solid lines are the parts of rough bark around knots. Bars = 5 cm.

The knots of *Abies* spp. are surrounded by rough texture (Fig 1). Bark surface textures (rough or not) were defined according to Toffin et al. [9]. We traced the distribution of the rough surface around knots on the bark (Fig 1) onto vinyl sheets. Then, we peeled the logs and recorded the distribution of entry holes by *P*. *proximus* on the vinyl sheets. We conducted these for three logs of each species from July 5 to July 8, and for the remaining two logs of each species from July 30 to August 2, 2019. The X-Y coordinates of entry holes were taken from the vinyl sheets, with the X-axis corresponding to the positions around the circumference of the tree, while the Y-axis corresponded to the positions along the length of the tree. To obtain the coordinates of the rough surface on the bark, the photographs of each vinyl sheet were uploaded to ArcGIS 10.6 [29] and the positions of rough bark were saved to shape-files for each log.

### Phloem thickness

We cut down two *A*. *veitchii* trees at Yatsugatake Forest Station, Mountain Science Center, University of Tsukuba, in Minamisaku County, Nagano Prefecture, Japan on July 7, 2020, and immediately peeled the bark. We randomly selected 18 knots and measured the phloem thickness of rough bark around the knots and that of the remaining bark near the knots [30].

### Statistical analysis

To determine the distribution pattern of entry holes, we carried out a K(*r*) function analysis using R 3.6.1 [31] with the “spatstat” package [32]. The K(*r*) function is a tool for analyzing spatial point process data [33]. K(*r*) is defined as the expected number of entry holes within distance *r* from a randomly chosen entry hole divided by the number of entry holes and attack density. To remove the effects of the cut-ends, the areas within 20 cm from both cut-ends were removed. Fifteen logs with 20 or more entry holes were used for the analysis. We applied Ripley’s edge correction, which is a method to correct the K(*r*) value by weighted value defined as the proportion of a circumference within the study area in the circumference of a circle centered at one point and passing through another point [34–36]. If a point pattern is completely spatially random (CSR) following a Poisson distribution, Ripley’s K(*r*) = π*r*^2^ [32,34,35]. The 95% confidence envelopes of observed K(*r*) values with Ripley’s edge correction were estimated from 10,000 simulations. The median of expected K(*r*) values with Ripley’s edge correction under the CSR were also estimated from 10,000 simulations by randomly generating entry holes. Since the gallery system of *P*. *proximus* consists of 2–3 egg galleries of 3–7 cm, which are generally oriented horizontally [37], the horizontal length of one gallery system is estimated at most about 14 cm. We, therefore, performed the K(*r*) function up to *r* = 15 cm. When the 95% confidence envelopes of observed K(*r*) values were larger or smaller than the median of the expected K(*r*) values under the CSR, the distribution pattern of entry holes was statistically significantly aggregated or uniform at the distance *r*, respectively.

To determine the effects of bark texture on the number of entry holes, we used generalized linear mixed models (GLMMs) with a Poisson distribution and a logarithm link, separately for each *Abies* species. The number of entry holes was the response variable, surface characteristics (i.e., the rough surface around knots or not) was an explanatory variable, the area of each surface characteristic was an offset term, and the log was a random effect. P-values were calculated by Wald chi-square tests and corrected using the Holm-Bonferroni method.

To determine the spatial association between entry holes and the rough surface around knots, we carried out a K_12_(*r*) function analysis (cross K-function analysis). The K_12_(*r*) function is a generalization of the K(*r*) function to a bivariate point process [38]. K_12_(*r*) is defined as the expected number of entry holes (= 1) within distance *r* from the boundary of a randomly chosen rough bark (= 2) divided by the number of rough bark and the attack density. Entry holes located in rough bark were considered to have a distance of 0. To remove the effects of the cut-ends, the areas within 20 cm from both cut-ends were removed from the analyses, while the rough bark surfaces around knots located in the remaining areas even a bit were used for the analyses. Fifteen logs with 20 or more entry holes were used for the analysis. We applied toroidal edge correction, where the original part was duplicated and the edge on one side was thought of as being wrapped around to the opposite edge [35,36]. We measured the distance from the boundary of the rough bark surface in the original part to entry holes both in the original and duplicated parts, and calculated K_12_(*r*) values at 0.5-cm intervals up to 15 cm using ArcGIS and R. The 95% confidence envelopes of the observed K_12_(*r*) value with toroidal edge correction was estimated from 10,000 simulations. The median of expected K_12_(*r*) values with toroidal edge correction under the CSR was also estimated from 10,000 simulations by randomly sampling the same number of knots as the observed number with replacement. When the 95% confidence envelopes of the observed K_12_(*r*) values were larger or smaller than the median of the expected K_12_(*r*) values under the CSR, the spatial association (aggregation/segregation) between the entry holes and rough surface was statistically significant at a distance *r*.

To determine if there was a significant difference in phloem thickness under the rough bark around the knots relative to phloem thickness under the remaining bark, we used the Wilcoxon rank-sum test.

## Results

**Fig 2:**
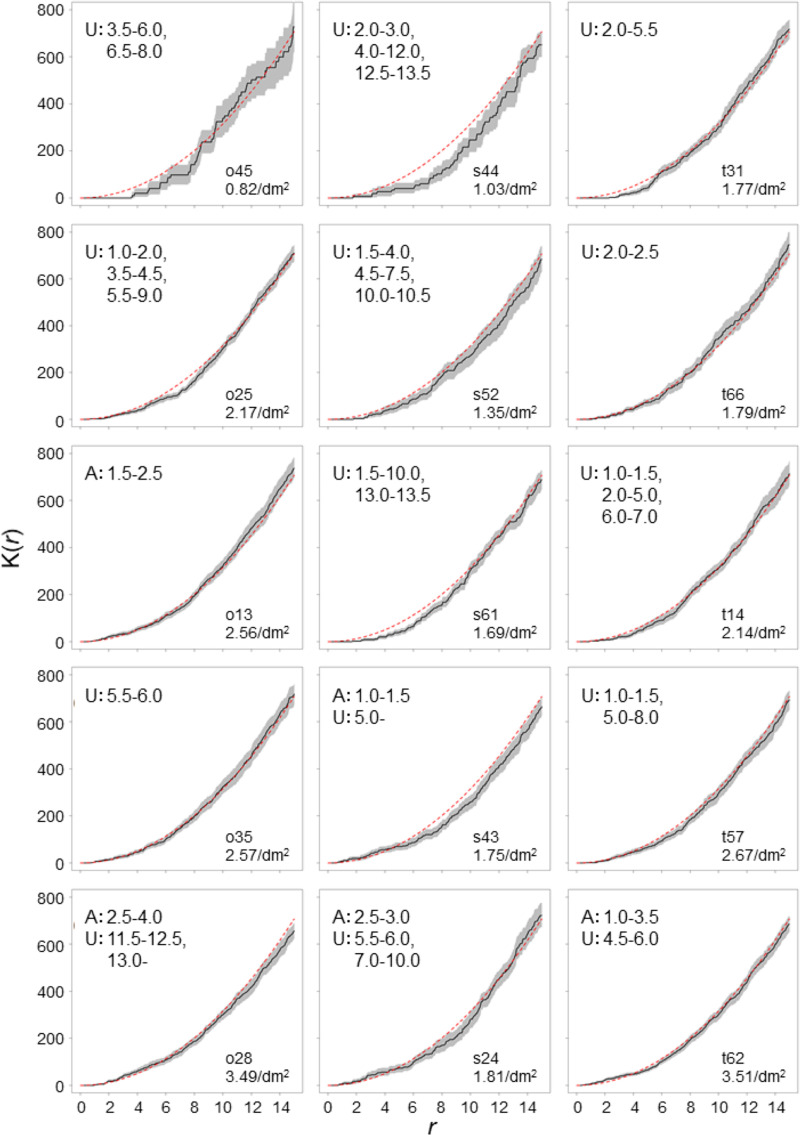
K(*r*) values calculated from spatial distribution of entry holes of *P*. *proximus*. The solid lines are K(*r*) values of the observed pattern, and the gray shaded areas are the 95% confidence envelopes estimated from 10,000 simulations. Red dashed lines are the median of estimated K(*r*) values under the completely spatially random pattern. The letters A and U indicate that significantly aggregated and uniform distribution patterns are observed, respectively, and the numbers following the letters indicate *r* ranges where the significance was observed.

The number of entry holes of each log ranged from 1 to 94 (Table 2, S1–S5 Figs), and 15 logs had 20 or more entry holes. Of the 15 logs, 10 logs exhibited a significantly uniform distribution pattern, and four logs demonstrated both uniform and aggregated distribution patterns, while one log exhibited a significantly aggregated distribution pattern of entry holes in the range of 1.0 cm to 4.0 cm (Fig 2). The five logs that demonstrated a significantly aggregated distribution pattern had a higher attack density in each species.

**Table 2:** Numbers of entry holes and areas of each bark texture of each log.

Species	Log ID	No. of entry holes in whole study part	Rough bark		Remaining bark	
			No. (density (/ dm^2^)) of entry holes	Area (dm^2^)	No. (density (/ dm^2^)) of entry holes	Area (dm^2^)
*A*. *firma*	m23	5	1 (0.22)	4.51	4 (0.16)	24.61
	m33	2	1 (0.22)	4.59	1 (0.04)	27.26
	m44	2	2 (0.86)	2.32	0 (0)	24.02
	m81	17	3 (0.9)	3.33	14 (0.46)	30.44
	m84	8	1 (0.38)	2.66	7 (0.24)	29.32
*A*. *mariesii*	o13	71	18 (9.33)	1.93	53 (2.06)	25.78
	o25	53	23 (8.68)	2.65	30 (1.38)	21.72
	o28	74	33 (10.96)	3.01	41 (2.25)	18.19
	o35	70	25 (11.19)	2.23	45 (1.79)	25.15
	o45	25	8 (1.34)	5.96	17 (0.7)	24.36
*A*. *veitchii*	s24	49	7 (4.77)	1.47	42 (1.64)	25.67
	s43	56	28 (7.38)	3.79	28 (1)	28.14
	s44	30	15 (4.56)	3.29	15 (0.58)	25.71
	s52	48	10 (2.01)	4.97	38 (1.24)	30.69
	s61	53	19 (5.85)	3.25	34 (1.21)	28.12
*A*. *sachalinensis*	t14	69	13 (6.09)	2.14	56 (1.86)	30.04
	t31	52	6 (2.81)	2.13	46 (1.68)	27.31
	t57	62	21 (10.75)	1.95	41 (1.93)	21.23
	t62	94	14 (9.54)	1.47	80 (3.16)	25.35
	t66	44	14 (5.52)	2.54	30 (1.36)	22.01
*A*. *homolepis*	u27	15	4 (2.62)	1.53	11 (0.54)	20.55
	u43	3	1 (0.29)	3.40	2 (0.07)	27.98
	u45	1	0 (0)	2.25	1 (0.04)	23.54
	u61	8	8 (1.28)	6.23	0 (0)	28.00
	u64	1	0 (0)	4.55	1 (0.04)	24.77

Attack density on the rough barks ranged from 0 to 11.19 / dm^2^, while that on the remaining parts ranged from 0 to 3.16 / dm^2^ (Table 2). The rough bark had significantly more entry holes than the remaining parts of bark for all species (χ^2^ = 5.79, P = 0.016 for *A*. *firma*; χ^2^ = 28.4, P < 0.001 for *A*. *homolepis*; χ^2^ = 165.1, P < 0.001 for *A*. *mariesii*; χ^2^ = 81.3, P < 0.001 for *A*. *sachalinensis*; χ^2^ = 109.1, and P < 0.001 for *A*. *veitchii*).

**Fig 3:**
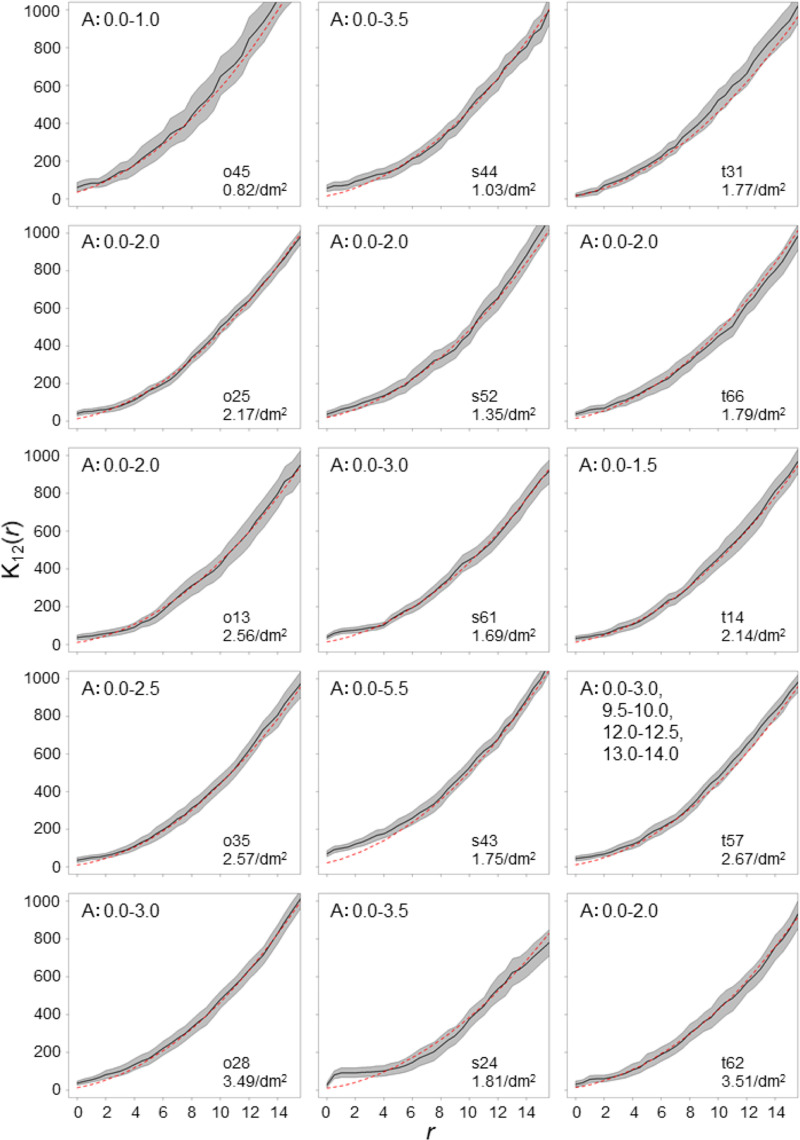
K_12_(*r*) values calculated from spatial association between entry holes and rough surface around knots. The solid lines are K_12_(*r*) values of the observed pattern, and the gray shaded areas are the 95% confidence envelopes estimated from 10,000 simulations. Red dashed lines are the median of estimated K_12_(*r*) values under the completely spatially random pattern. The letter A indicates that significantly aggregated distribution patterns are observed, and the numbers following the letter indicate *r* ranges where the significance was observed.

Of the 15 logs, which had 20 or more entry holes, 14 logs revealed that entry holes had significantly aggregated spatial association with rough bark in the range of 5.5 cm, while one log did not reveal significant spatial association with rough bark (Fig 3).

**Fig 4:**
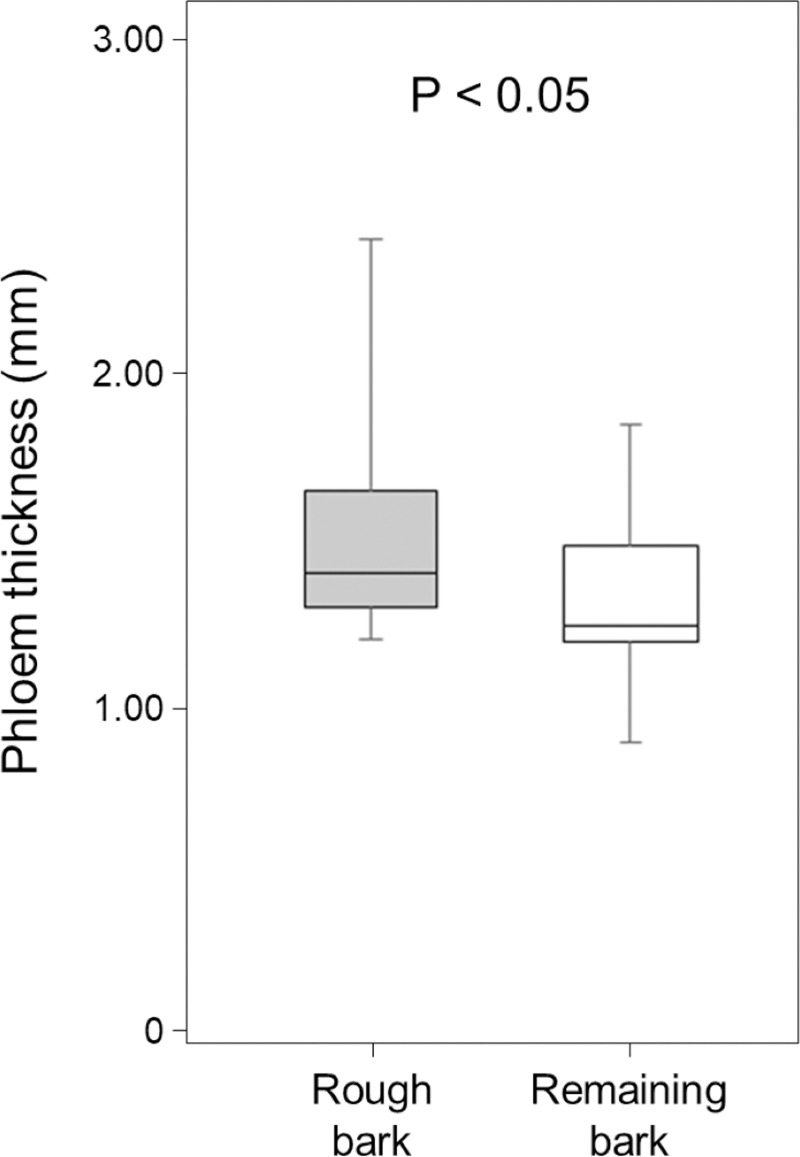
Phloem thickness of rough bark around knots and remaining bark near knots.

Rough bark around knots had significantly thicker phloem than the remaining parts of bark (Wilcoxon rank-sum test; W = 225, P < 0.05, Fig 4).

## Discussion

The present study revealed the distribution pattern of the bark beetle *P*. *proximus*. Our results demonstrated that the distribution pattern of entry holes of *P*. *proximus* was significantly uniform in most cases. Numerous studies have demonstrated that the distribution pattern of bark beetle entry holes is uniform to avoid intraspecific competition [4,5]. The uniform distribution pattern of entry holes observed in the present study suggests that *P*. *proximus* adults avoid other entry holes when they attack.

However, the distribution pattern of entry holes was aggregated within 4.0 cm when the attack density was higher. The K_12_(*r*) values revealed that the distribution of entry holes was significantly aggregated within 5.5 cm from rough bark around knots in most cases. The GLMMs also revealed that rough bark had significantly more entry holes than the remaining bark. These results indicate that the bark surface texture affects the selection of the entry point of *P*. *proximus*. These also suggest that the increase in attack density on the rough bark around knots may result in the aggregated distribution of entry holes.

Previous studies have demonstrated that bark texture plays a strong role in the choice of attack location [8,9,39]. The attack density of several bark beetles is higher in rough bark. For example, *Ips typographus* revealed a higher attack density around knots than that on the remaining part of the bark [9]. The mechanism underlying the aggregated distribution in rough bark is still unclear. Ferrenberg and Mitton [8] suggested that smooth textured-bark acts as an anatomical defense against the bark beetle *Dendroctonus ponderosae* by reducing their ability to grip a tree surface, resulting in a higher attack density on rough barks such as cracks, flakes, and crenulations. We revealed that rough bark around knots has thicker phloem than the remaining barks. Phloem thickness also plays a strong role in reproductive success and larval performance [40–42]. Our results suggest that *P*. *proximus* may preferentially attack rough bark to reproduce in the thicker phloem under the rough bark surface. To determine whether *P*. *proximus* preferentially attacks rough bark or avoids smooth bark, choice tests should be conducted.

A higher attack density results in severe intraspecific competition for nutritious phloem among larvae and low reproductive success [1–3]. The higher attack density in rough bark around knots may eventually cause severe intraspecific competition. Further studies on the differences in reproductive potential and fecundity of females depending on bark thickness should be conducted.

## Supporting information

S1 Fig Distribution of entry holes (red points) and rough bark around knots (shaded areas) on *A*. *firma* log surfaces where areas within 20 cm from cut-ends were removed. The letters and numeric indicate log ID. Bar = 10 cm.
(PDF)

S2 Fig Distribution of entry holes (red points) and rough bark around knots (shaded areas) on *A*. *mariesii* log surfaces where areas within 20 cm from cut-ends were removed. The letters and numeric indicate log ID. Bar = 10 cm.
(PDF)

S3 Fig Distribution of entry holes (red points) and rough bark around knots (shaded areas) on *A*. veitchii log surfaces where areas within 20 cm from cut-ends were removed. The letters and numeric indicate log ID. Bar = 10 cm.
(PDF)

S4 Fig Distribution of entry holes (red points) and rough bark around knots (shaded areas) on *A*. *sachalinensis* log surfaces where areas within 20 cm from cut-ends were removed. The letters and numeric indicate log ID. Bar = 10 cm.
(PDF)

S5 Fig Distribution of entry holes (red points) and rough bark around knots (shaded areas) on *A*. *homolepis* log surfaces where areas within 20 cm from cut-ends were removed. The letters and numeric indicate log ID. Bar = 10 cm.
(PDF)
