# Supplementary material for: Distribution pattern of entry holes of the tree-killing bark beetle Polygraphus proximus
Source: PLoS One. 2021 Feb 9;16(2):e0246812. doi: 10.1371/journal.pone.0246812 (PMC7872293; doi:10.1371/journal.pone.0246812)
Supplement: S1 Fig — The letters and numeric indicate log ID. Bar = 10 cm. (PDF) [file pone.0246812.s001.pdf]

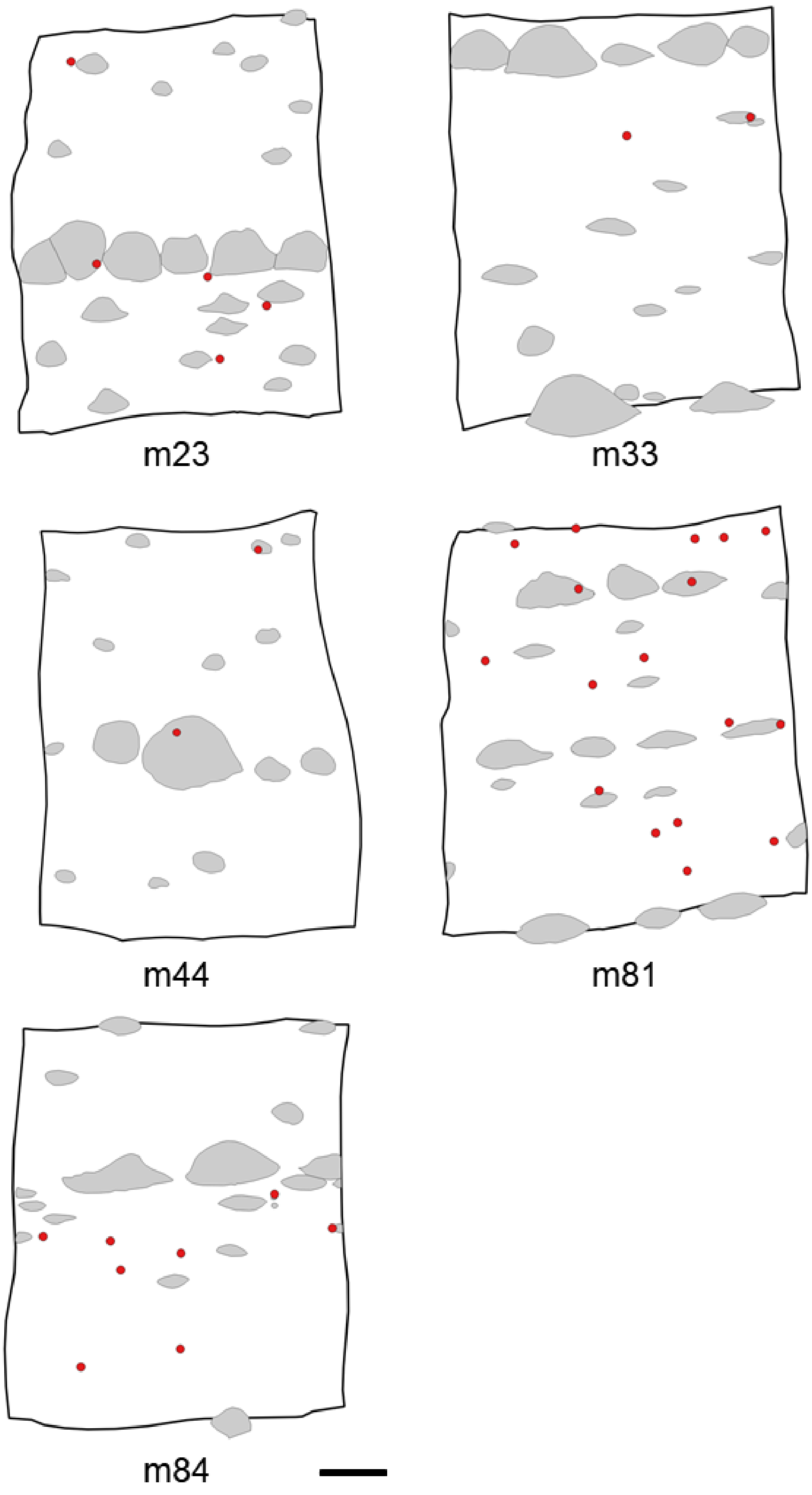

S1 Fig. Distribution of entry holes (red points) and rough bark around knots (shaded areas) on *A. firma* log surfaces where areas within 20 cm from cut-ends were removed. The letters and numeric indicate log ID. Bar = 10 cm.
